# Supplementary material for: Healthcare resource utilization and associated costs among patients with migraine in Finland: A retrospective register-based study
Source: PLoS One. 2024 Mar 20;19(3):e0300816. doi: 10.1371/journal.pone.0300816 (PMC10954127; doi:10.1371/journal.pone.0300816)
Supplement: S1 Table — (DOCX) [file pone.0300816.s001.docx]

**S1 Table. Triptane contraindication and migraine preventive medication definitions**

|  | ICD-10 codes | ATC codes |
| --- | --- | --- |
| **Triptane contraindications**  (Comorbidities data from THL HILMO and AvoHILMO registries) | I20-I25  I63-I64  I71-I72  I74  I77  I79  G45  I11-I5  K72  K74 |  |
| **Migraine preventive medication**  (Prescription data from THL HILMO and AvoHILMO registries) |  | C09CA06 Candesartan  C07AA05 Propranolol  C07AB02 Metoprolol  C07AB07 Bisoprolol  C08DA01  Calcium channel blockers  N06AA09  Amitriptyline  N03AX11 Topiramate  N03AG01 Valproate  N06AX16  Venlaflaxine  N03AX09  Lamotrigine |
