# Supplementary material for: Healthcare resource utilization and associated costs among patients with migraine in Finland: A retrospective register-based study
Source: PLoS One. 2024 Mar 20;19(3):e0300816. doi: 10.1371/journal.pone.0300816 (PMC10954127; doi:10.1371/journal.pone.0300816)
Supplement: S4 Table — (DOCX) [file pone.0300816.s004.docx]

**S4 Table. The breakdown of migraine related contacts into different HCRU components**


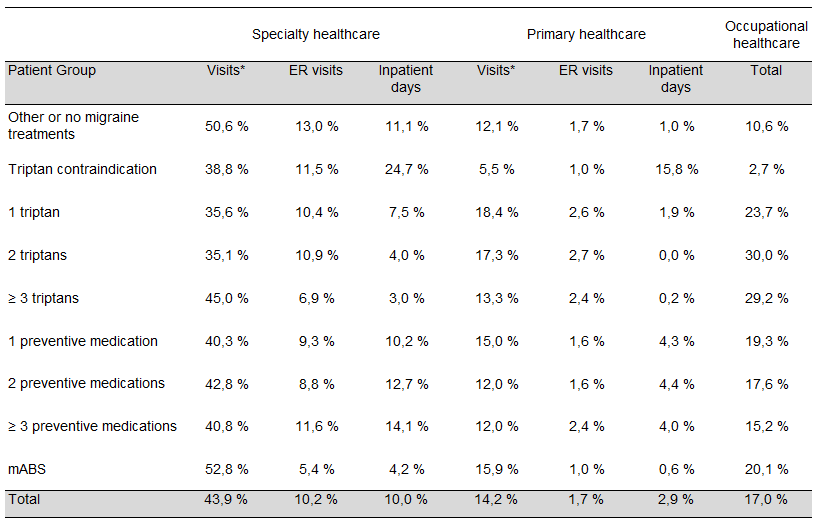


*Other than emergency room (ER) visits & other than inpatient days.
