# Supplementary figures and images for: Healthcare resource utilization and associated costs among patients with migraine in Finland: A retrospective register-based study
Source: PLoS One. 2024 Mar 20;19(3):e0300816. doi: 10.1371/journal.pone.0300816 (PMC10954127; doi:10.1371/journal.pone.0300816)

**S5 Table. The breakdown of migraine related costs into different HCRU components**


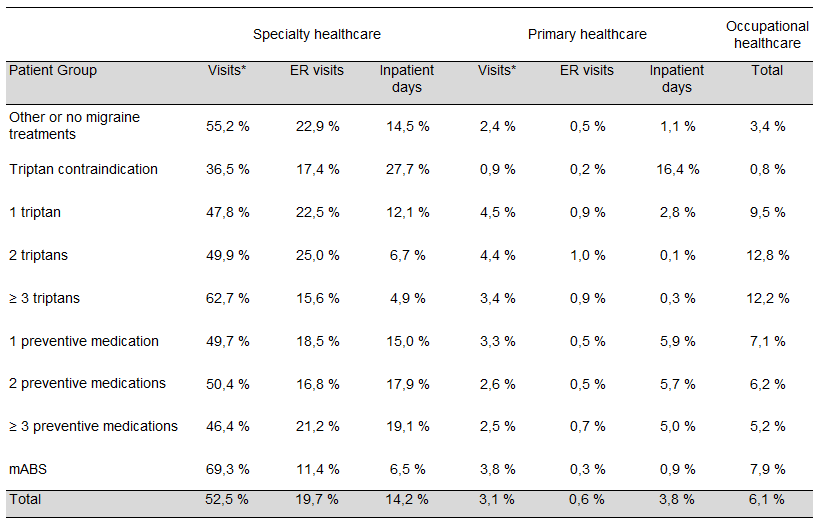

Supplement: S5 Table — (DOCX) [file pone.0300816.s005.docx]
