# Supplementary material for: Healthcare resource utilization and associated costs among patients with migraine in Finland: A retrospective register-based study
Source: PLoS One. 2024 Mar 20;19(3):e0300816. doi: 10.1371/journal.pone.0300816 (PMC10954127; doi:10.1371/journal.pone.0300816)
Supplement: Attachment — Submitted filename: Response to reviewers.docx [file pone.0300816.s007.docx]

The authors’ comments can be found in blue below.

**Reviewer #1:**

The title is very appropriate, and the summary section covers the central aspect of the study. The introduction provides background and information relevant to the research and is very carefully written. The methods are transparent and replicable; all results match the described methods. While the results are not new, they represent progress in this area. The data is plausible. The findings described by the author correlate with the results, and the findings are relevant. The conclusions correlate to the results found. The authors have provided 5 figures and 2 tables; all are clear and legible. I think this article is precious and it would be useful to publish it in your journal.

Dear Reviewer,

We would like to express our sincere gratitude for your thoughtful review of our manuscript. Your kind words are greatly appreciated, and we acknowledge your time and effort in providing us with your comments. We feel that our manuscript will bring novel value in assessing the real-life burden of migraine in Finland and in bringing much needed insights for patients and decision makers.

**Reviewer #2:**

The authors present a retrospective, descriptive analysis of healthcare costs in patients diagnosed with or treated for migraines in a Finnish registry. The authors present two major conclusions: that healthcare costs associated with migraines are significant and likely correlated with disease severity, and that an earlier threshold for novel treatments may be warranted.

Dear Reviewer,

We greatly appreciate your thoughtful and through review of our manuscript, and your time and effort in providing constructive feedback that will help to improve the quality of our research. Your comments have been invaluable in shaping the final version of our manuscript, and we are grateful for the time and effort you have invested in this process. We have taken your feedback in consideration and made the necessary revisions to improve the quality of our work.

The first conclusion is reasonably well-supported by the authors' findings and is consequential to policy-makers in understanding resource utilization for migraines. One methodological question that could be further clarified relates to patients receiving preventive medications--the authors appear to identify prescriptions for antihypertensives, certain anticonvulsants, amitriptyline, and venlafaxine as preventive treatments for migraines within two weeks of a migraine billing code, although there does not appear to be an analysis of how many of these encounters (or patients) also had billing codes for hypertension, seizures, or mood disorders that could have influenced these prescriptions. Since these medications may well have been selected to address both migraines and a systemic comorbidity, these data points need not necessarily be eliminated, but further insights into potential confounding comorbidities would help the reader better understand the group requiring 3+ preventive medications, which is responsible for disproportionately high migraine-related and all-cause healthcare costs. Compared to the cohorts receiving triptans alone, the groups receiving preventive medications generally appear to have a greater proportion of patients aged 65+, which could again point to confounding from systemic comorbidities.

We acknowledge the valid point raised by the reviewer. Unfortunately, we do not have data on comorbidities by different migraine patient groups available for our study. Thus, this highlights the additional need to provide further input on this topic. To our understanding the largest difference in the results regarding the proportion of patients aged 65+ compared to other groups is eminent in the triptan contraindication group. The share of patients aged 65+ is somewhat lower in the 2 and 3 triptans group compared to all preventive medication groups, but not compared to 1 triptan group which represented the largest population of the triptan treated patients. We have taken the reviewers feedback into account and included additional discussion on the topic of cofounding comorbidities into the study limitations within the Discussion section as follows:

*“A study with migraine patients in Finland demonstrated that the burden of migraine increased by failed treatment lines and was associated with increased comorbidity, with a positive correlation of treatment lines and frequency of comorbidities [16]. The study also showed that in patients with 3 or more prophylactic treatments, a mental disorder such as major depressive disorder or anxiety, was diagnosed in every third person. No data on diagnosis specific medicine purchases are available from the registries included in this study. Thus, in order to reduce the potential confounding of different comorbidities to medicine prescriptions, a medication prescription was tied to a migraine diagnosis related healthcare visit with no more than two weeks prior each prescription. But as our study does not include data on comorbidities within different migraine patient groups, we cannot exclude the possibility that comorbidities such as hypertension, seizures, or mood disorders have not influenced the prescriptions, and consequently the formation of the patient groups. Nevertheless, the mean age and sex distribution of patients treated with acute or prophylactic medications in our study is similar with the previous study of patients with migraine in Finland [16]. Our study also shows similar trends in all-cause, and migraine related healthcare visits presented in that study, demonstrating an increasing burden by each failed treatment and showing the highest burden for patients requiring ≥ 3 preventive medications.”*

The supporting evidence for the authors' second assertion regarding earlier use of novel therapies is less clear. The authors rightfully note the "unmet need in patients who have had inadequate response to two or more triptans" and show a modest increase in migraine-related healthcare costs in patients receiving multiple triptans but omit a discussion regarding the very germane costs of CGRP antagonists as acute therapies for migraines. While these data are limited in episodic migraine, prior studies have suggested cost-effectiveness in chronic migraines only with steeply discounted drug prices (PMID 31302899) or in cohorts that had not responded to multiple prior treatments (PMID 36114468), which would weigh against early use of novel therapies. Moreover, the utility of CGRP antagonists in patients with contraindications to triptans (which had the second-largest healthcare expenditures in the study) remains uncertain given unresolved questions about the effects of CGRP blockade in ischemia. Should the authors wish to include a discussion on the role of novel therapies in episodic migraines, a more nuanced discussion of cost-effectiveness and an additional review of the literature would be appropriate.

We highly appreciate the reviewer’s comments and have now further extended the discussion of the unmet need in migraine in the context of cost-effectiveness of novel treatments options in the revised manuscript. Indeed, CGRPs in Finland are reimbursed with restrictions in the preventive treatment of migraine to patients who have tried at least two anti-migraine medications that have not produced an adequate response, are contraindicated or not tolerated. We agree that the topic of cost-effectiveness enhances the discussion section of our study and brings additional insights to the second conclusion of our manuscript, which will also be rephased. We have extended the discussion as the reviewer suggested and have included the references highlighted by the reviewer. The following text to the Discussion section of our study:

*“Our study demonstrated that migraine related HCRU and associated costs increased relatively in the acute treatment group and in the preventive medications group for patients receiving multiple prior medications, with patients with three or more preventive medications having the highest total migraine related HCRU and costs of the studied patient cohorts. Migraine related HCRU and costs were also relatively high for patients with triptan contraindications. As new treatment options, such as CGRPs have been shown to provide favorable health outcomes to patients in clinical studies, they are also associated with higher medicine costs compared to triptans or anti-migraine medications used in* preventive care.

*Prior studies have suggested CGRPs to be cost-effective in chronic migraine only with highly discounted drug prices [23] or in cohorts that had not responded to multiple prior treatments [24]. In Finland CGRPs are reimbursed with restrictions and confidential price agreements for prophylactic migraine to patients who have an average at least 8 migraine days per month when initiating treatment, and who have tried at least two anti-migraine medications that have not produced an adequate response, are contraindicated or not tolerated. Furthermore, the eligibility for reimbursement is evaluated periodically based on the reduction of monthly migraine days. To analyze the cost-effectiveness of treatments in each healthcare system it is important to consider the local burden of disease when evaluating which patients could benefit the most from novel treatments, and where potential savings with new treatment options within the healthcare system could be achieved.”*

We have rephrased the second assertion in the conclusion section to be as follows:

*“**In many healthcare systems resources are currently strained and the need for different healthcare services is most likely increasing due to the aging population. When assessing the patient access and cost-effectiveness of novel treatments for the treatment of migraine within different healthcare systems, a holistic analysis of the current disease burden along with potential gains achieved for patients and healthcare service providers are essential information in guiding decision-making. Nevertheless, the novel treatments targeting CGRP-pathway represent a promising treatment option, especially in those patients previous acute- or preventive migraine medication has failed. In future, studies on the cost-effectiveness of CGRP-pathway targeting medications in the treatment of episodic migraine are warranted.”*

We also reflected the above Conclusion within the Abstract:

*“When assessing the patient access and cost-effectiveness of novel treatments for the treatment of migraine within different healthcare systems, a holistic analysis of the current disease burden along with potential gains for patients and healthcare service providers are essential information in guiding decision-making.”*

In terms of minor revisions:

Line 52 - could be reworded for clarity (e.g., "noted an average of 5.9 doctor's visits per month over the preceding six months")

We appreciate the suggestion for rewording. This change has been made to the manuscript.

Line 77 - "attack" may be a more recognizable term to readers than "seizure"

Noted and the change has been made to the manuscript.

Line 87 - blockade of the renin-angiotensin system appears to apply to antihypertensives rather than anticonvulsants, including in the listed reference

We thank the reviewer for this remark, the sentence has been modified as follows*:*

*“Preventive medication is used to reduce the frequency, severity, and duration of migraine attacks, and include drugs such as beta and potassium blockers, tricyclic antidepressants, antiepileptics, and drugs affecting the renin-angiotensin system”.*

Line 132 - "giant cell arteritis" might be recognizable to a broader audience than "Horton disease"

We thank the reviewer for this observation. In Finland ICD-10 code G44.0 diseases are often referred as Horton disease or Horton headache. We acknowledge that this local wording may not be clear for a broader audience, and we have changed Hortons disease in the text to “Cluster headaches” in line with the ICD-10 code G44.0. We have also changed Hortons diseases to “cluster headaches” in figure 1.

Line 410 - as noted above, this observation in patients with likely chronic migraines may not apply to those with episodic migraines treated with triptans; additionally, it would be difficult to infer that CGRP antagonists led to a reduction in healthcare costs as the remaining patients on multiple preventive agents do not represent an adequate control group.

As mentioned within our comment on the second assertion above, we have modified our Conclusion section and Abstract of the manuscript referring to this revision suggestion as follows:

Conclusion section:

*“In many healthcare systems resources are currently strained and the need for different healthcare services is most likely increasing due to the aging population. When assessing the patient access and cost-effectiveness of novel treatments for the treatment of migraine within different healthcare systems, a holistic analysis of the current disease burden along with potential gains achieved for patients and healthcare service providers are essential information in guiding decision-making. Nevertheless, the novel treatments targeting CGRP-pathway represent a promising treatment option, especially in those patients previous acute- or preventive migraine medication has failed. In future, studies on the cost-effectiveness of CGRP-pathway targeting medications in the treatment of episodic migraine are warranted.”*

Abstract:

*“When assessing the patient access and cost-effectiveness of novel treatments for the treatment of migraine within different healthcare systems, a holistic analysis of the current disease burden along with potential gains for patients and healthcare service providers are essential information in guiding decision-making.”*

**Additional Sources added to the manuscript.**

[23] Giannouchos TV, Mitsikostas D-D, Ohsfeldt RL, Vozikis A, Koufopoulou P. Cost‑Effectiveness Analysis of Erenumab Versus OnabotulinumtoxinA for Patients with Chronic Migraine Attacks in Greece. Clinical Drug Investigation. 2019;39: 979–990. doi:10.1007/s40261-019-00827-z

[24] Khanal S, Underwood M, Naghdi S, Brown A, Duncan C, Matharu M, et al. A systematic review of economic evaluations of pharmacological treatments for adults with chronic migraine. The Journal of Headache and Pain. 2022;23:122. doi:10.1186/s10194-022-01492-y
